# Inverted CdSe/PbSe Core/Shell Quantum Dots with Electrically Accessible Photocarriers

**DOI:** 10.1021/acsenergylett.4c03502

**Published:** 2025-02-05

**Authors:** Vladimir Sayevich, Whi Dong Kim, Zachary L. Robinson, Oleg V. Kozlov, Clément Livache, Namyoung Ahn, Heeyoung Jung, Victor I. Klimov

**Affiliations:** Nanotechnology and Advanced Spectroscopy Team, C-PCS, Chemistry Division, Los Alamos National Laboratory, Los Alamos, New Mexico 87545, United States

## Abstract

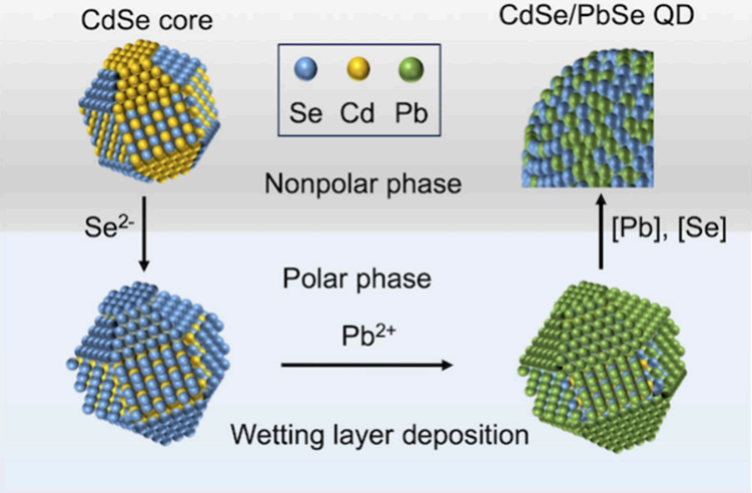

Heterostructured quantum dots (QDs) based on narrow-bandgap PbSe and wide-bandgap CdSe have been studied for applications in near-infrared light sources, photodetection, and solar energy conversion. A common structural motif is a QD consisting of a PbSe core enclosed in a CdSe shell. However, the CdSe shell complicates extraction of band-edge charge carriers from the QD. Therefore, conventional PbSe/CdSe QDs are not suitable for application in photoelectric devices. Here we report inverted CdSe/PbSe core/shell QDs that overcome this drawback. In these structures, both the electron and hole exhibit a significant degree of shell localization and can therefore be easily extracted from the QD. To create these structures, we employ a thin, atomically controlled wetting layer that homogenizes the CdSe core surface and thus promotes directionally uniform growth of the PbSe shell. The synthesized CdSe/PbSe QD films exhibit good photocarrier transport, making them suitable for application in photoelectric devices.

By combining different materials in a single nanocrystalline heterostructure, it is possible to realize properties not accessible with monocomponent systems. In the case of colloidal heteronanocrystals, or quantum dots (QDs), a common structural motive is a core/shell or a core/multishell QD.^1−4^ The core/shell approach allows manipulation of many QD functionalities, including the emission efficiency, spectral energy and lifetime,^1,5,6^ the character of the band-edge transition (direct versus indirect),^7,8^ the exciton–exciton interaction energy,^9^ the Auger recombination rate,^10−12^ and the emission intermittency of a single QD emitter.^13,14^

Current methods to synthesize core/shell structures are based on direct growth of one semiconductor on top of a core made of other material (additive growth) or anion/cation exchange within a peripheral layer of a presynthesized core. The ion exchange method has been often applied to prepare PbSe(S)/CdSe(S) core/shell QDs.^15−20^ These heterostructures have been extensively studied as high-efficiency near-infrared (NIR) light emitters.^21−23^ In principle, they are also attractive for application in photoelectric devices, including advanced systems exploiting carrier multiplication^24^ (CM) and up-conversion^25,26^ concepts. However, the realization of such devices with conventional core/shell PbSe/CdSe QDs is not straightforward since photogenerated carriers are predominantly confined to the PbSe core, which complicates their extraction from the QD due to a large potential barrier created by the CdSe shell. This barrier is especially high (∼1.4 eV) for photoholes,^17,24^ which makes them not easily accessible electrically. In particular, according to previous studies of PbSe/CdSe QDs, electron scavengers have a quenching effect on QD emission while hole scavengers do not, indicating that holes cannot be extracted from the QD.^19^

The problem of charge extraction can, in principle, be resolved by inverting the nanostructure, that is, by preparing CdSe(core)/PbSe(shell) QDs. In such inverted structures, wave functions of both an electron and a hole are expected to extend to the QD surface which should simplify their extraction from the QD. Inverted core/shell QDs, in which a higher bandgap semiconductor core is enclosed in a shell of narrow gap material, have been previously realized using mainly II–VI semiconductors.^6,27,28^ Inverted CdE_1_/PbE_2_ QDs (E_1_, E_2_ = S, Se) are less common due to various synthetic challenges that complicate their fabrication.^29^ The use of additive approaches to prepare spherically symmetric CdE_1_/PbE_2_ core/shell QDs is at least partially complicated by significant differences in the reactivity of the different crystal facets of a CdSe(S) core, compounded by inhomogeneities in ligand coverage.^30^ On the other hand, the application of traditional cation exchange usually results in either a complete replacement of Cd^2+^ for Pb^2+^ ions in the entire QD volume^31^ or asymmetric cation exchange^18^ often leading to segmented heterostructures such as Janus particles.^32,33^

To suppress undesirable cation exchange during additive growth of the PbS shell, the authors of ref^29^ used room-temperature deposition of the shell material, which allowed them to realize CdSe/PbS core/shell QDs. However, the synthesized structures did not exhibit NIR emission, probably due to the large number of crystal defects created during the room-temperature growth of the shell material.

Here we report a synthetic method, wetting-layer-assisted deposition (WLAD), that allows us to fabricate symmetric NIR emitting hetero-QDs with an inverted CdSe(core)/PbSe(shell) geometry. This method combines the advantages of atomic-level precision of a room-temperature colloidal atomic layer deposition^34^ (c-ALD) with the high crystalline quality of materials created via high-temperature synthesis. The key element of the WLAD technique is a thin atomically controlled wetting layer (WL) of PbSe prepared on top of a CdSe core using self-limited room-temperature c-ALD. This approach allows us to eliminate surface nonuniformities arising from a facet-dependent crystal structure and/or a nonuniform composition/density of a molecular ligand layer. By introducing the WL, we obtain a homogeneous, highly reactive core surface, which promotes directionally symmetric growth of a high-quality PbSe shell obtained by high-temperature additive growth. We show that the WLAD technique can be successfully applied to prepare inverted CdSe/PbSe core/shell structures using starting cores with both a wurtzite (WZ) and zinc-blende (ZB) crystal structure. The fabricated QDs exhibit dual-band photoluminescence (PL) comprising a visible and NIR emission features, as previously observed for conventional (noninverted) PbSe/CdSe core/shell QDs. Further, time-resolved photocurrent measurements reveal efficient hole-dominated charge transport which is similar to that of device-grade films of core-only PbSe(S) QDs. Thus, the developed inverted CdSe/PbSe core/shell QDs combine optical characteristics similar to those of conventional PbSe/CdSe QDs with good photocarrier transport properties derived from a favorable distribution of carrier wave functions that exhibit a considerable degree of shell localization.

The WLAD approach comprises two steps: (1) polar-phase-based deposition of a WL of a shell material (PbSe) onto presynthesized CdSe cores using room-temperature c-ALD,^34^ followed by (2) the high-temperature growth of a uniform highly crystalline PbSe shell in a nonpolar phase performed by adding controllable amounts of molecular precursors (Figure 1, Scheme S1, and Experimental Section in the Supporting Information). Briefly, first, we use established nonpolar-phase syntheses to prepare highly monodisperse CdSe cores with either WZ^35^ or ZB^36^ lattices (Figure 1a). The fabricated nanocrystals are transferred into a polar solvent (*N*-methylformamide, MFA), which is accompanied by the replacement of original organic ligands (mostly aliphatic alkylcarboxylates and phosphates) with Se^2–^ species (Figure 1b). Negatively charged Se^2–^ ionic ligands feature high binding affinity to electrophilic moieties on a CdSe QD surface, which helps remove all original organic molecules independent of the strength of their bonding to the nanocrystal surface and/or the attachment mode.^37^ In particular, as indicated by measurements using a Fourier transform infrared (FTIR) spectroscopy (Figure S1), FTIR features due to the C–H stretching modes are pronounced in the original samples. However, they completely disappear from the FTIR spectra following organic-for-inorganic ligand exchange.

**Figure 1:**
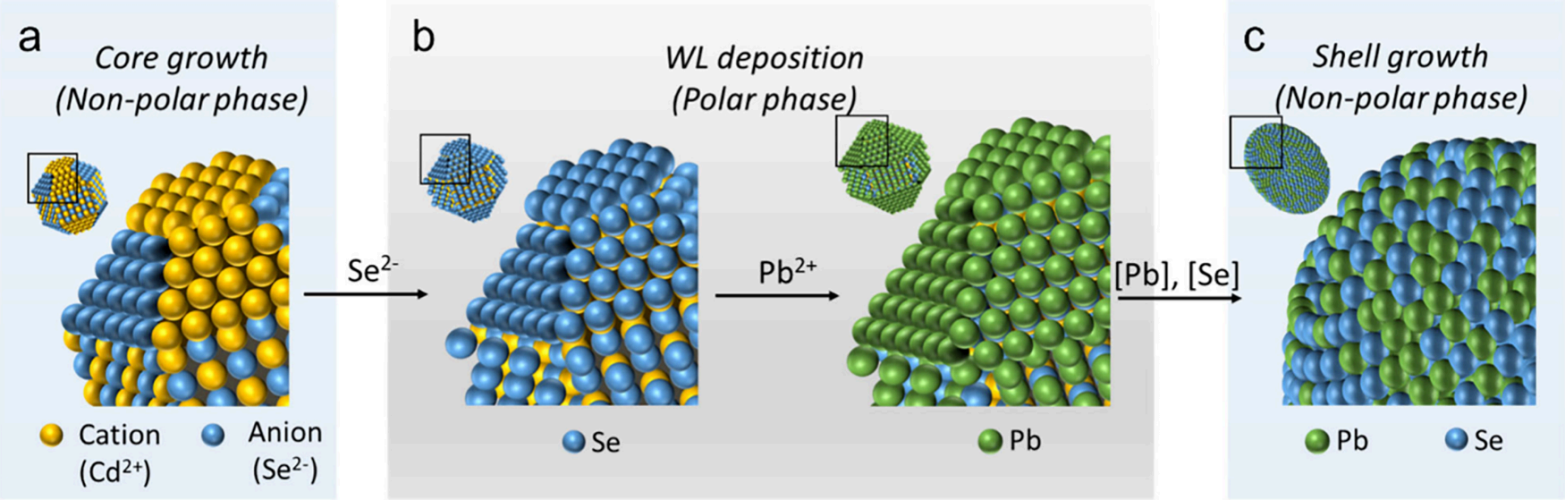
Schematic representation of the synthesis of inverted CdSe/PbSe core/shell QDs using the wetting-layer-assisted deposition (WLAD) method. (a) The first reaction step is the standard nonpolar-phase synthesis of CdSe cores with ZB or WZ crystal structures. (b) This is followed by transfer of the synthesized cores into a polar solvent, accompanied by the replacement of the original organic ligands with ionic Se^2–^ species. The Se^2–^ terminated QDs are then reacted with Pb^2+^ cations, resulting in the formation of a highly unform PbSe WL. (c) In the third step, the WL-capped QDs (CdSe+WL) are transferred into a nonpolar phase and reacted with controlled amounts of Pb and Se molecular precursors in the presence of oleate coligands to prepare a PbSe shell of the desired thickness.

After the Se^2–^-terminated CdSe cores are transferred into a polar solvent, they are reacted with Pb^2+^ using c-ALD, which leads to the formation of a Se–Pb WL (Figure 1b). This layer is highly uniform and comprises exactly one semiconductor monolayer (ML) of a targeted shell material. Due to small sizes of Se^2–^ and Pb^2+^ ions compared to the size of conventional organic ligands, the WL can easily adapt an irregular surface morphology of a faceted colloidal nanocrystal which helps homogenize the particle surface and thereby facilitate follow-up isotropic growth of the PbSe shell. The particles produced at this stage are further referred to as (CdSe+WL) functionalized cores.

To continue shell growth, we add lead(II) oleate to the Pb^2+^-terminated functionalized cores and transfer them back into a nonpolar phase (Figure 1c). “Soft” oleate anions feature good affinity to “soft” surface Pb^2+^ cations, which is combined with their high lability at elevated temperatures.^38^ The former property facilitates transfer of the particles into a nonpolar medium, while the latter simplifies simultaneous nucleation of PbSe at multiple sites over the entire nanocrystal surface which is essential for promoting uniform shell growth.^30,39^ Following transfer into a nonpolar solvent, we react the (CdSe+WL) structures with controlled quantities of precursors of Se (trioctylphosphine selenide, TOP-Se) and Pb (lead oleate, Pb-OL or lead chloride oleylamine, PbCl_2_·OLA) to achieve a desired shell thickness (Figure 1c and Scheme S1).^40^ The amounts of Pb and Se stock solutions were calculated from the core and shell volumes using bulk lattice parameters of PbSe and CdSe.

To grow the shell we prepare two syringes, one of which is filled with thoroughly washed functionalized (CdSe+WL) cores dissolved in octadecene (ODE) and another with the solution of the Se-precursor. Then, we inject the contents of both syringes into a mixture of the Pb precursor, ODE, and oleic acid (OA) heated to temperature *T* = 130–140 °C. Afterward, the temperature of the reaction mixture is raised to 150 °C for the ZB cores or to 160 °C for the WZ cores. The reaction is allowed to proceed for 0.5–4 min and then quenched by fast cooling. The longer reaction time leads to a thicker PbSe shell. This method allows us to grow the PbSe shell with a thickness (*H*) of up to ∼1.2 nm, which corresponds to ∼4 semiconductor MLs. Thicker PbSe shells were obtained by additional injections of Pb- and Se-precursors into the solution of the CdSe/PbSe QDs at 140 °C (Scheme S1).

Transmission electron microscopy (TEM) images of the initial ZB CdSe cores and the final inverted core/shell CdSe/PbSe QDs are shown in Figure 2a. The starting CdSe cores are characterized by a mean radius (*r*) of 2 nm and a standard size deviation (δ*r*) of 0.14 nm, which corresponds to δ*r*/*r* of ∼7% (Figure 2b, blue). Following the deposition of the PbSe shell, the particle radius increases to *R* = 2.9 nm, and the size dispersion (δ*R*/*R*) broadens to ∼14% (Figure 2b, red). Using the core and core/shell QD sizes, we find that the average shell thickness is *H* = 0.9 nm or ∼3 PbSe MLs. Based on the analysis of size polydispersities (δ*r* and δ*R*), the standard deviation of shell thickness (δ*H*) is ∼0.38 nm or ∼1 ML.

**Figure 2:**
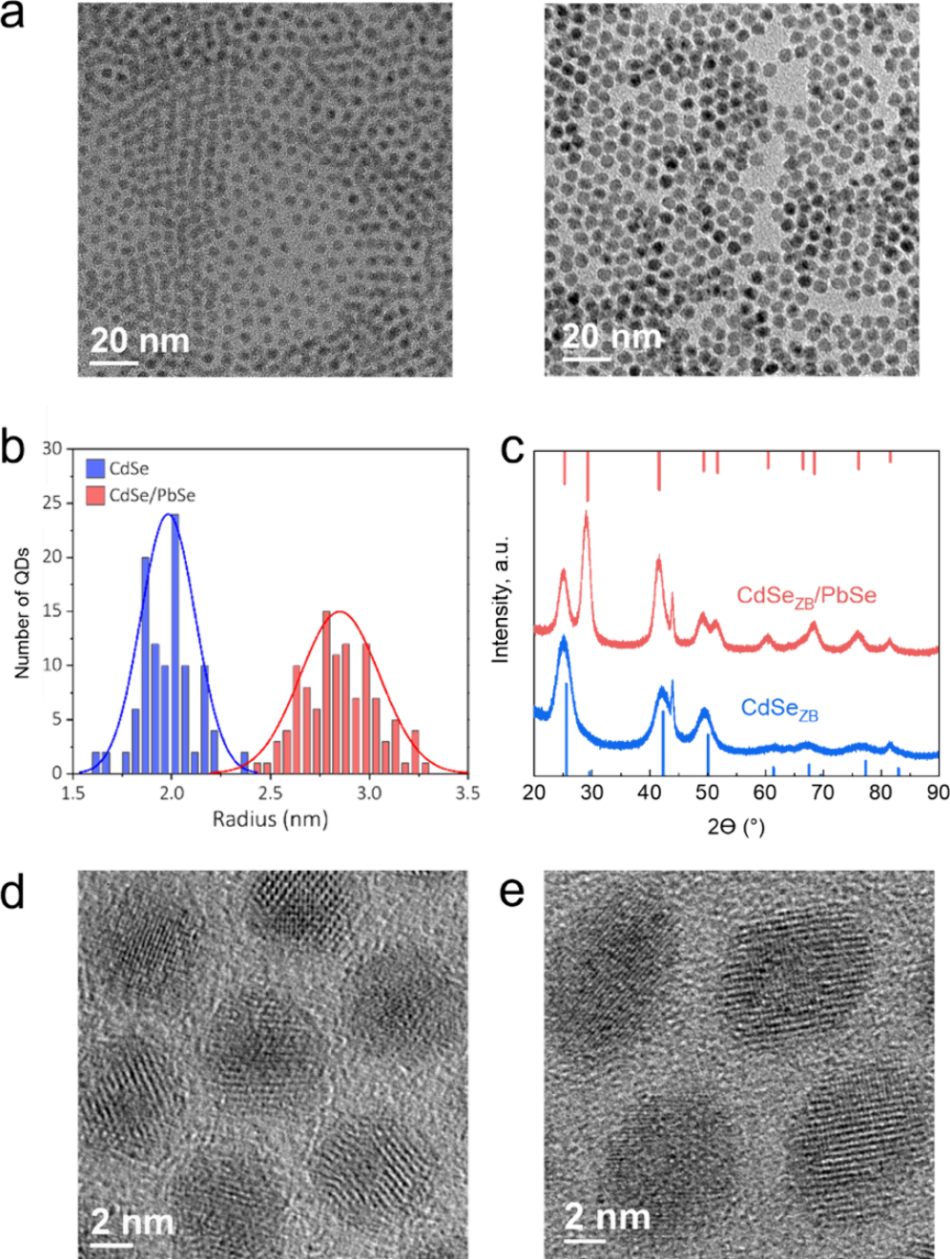
(a) TEM images of the starting ZB CdSe cores (left) and the final core/shell CdSe/PbSe QDs (right). (b) Size distributions of the ZB CdSe cores (blue bars) and final core–shell QDs (red bars). Based on a Gaussian fit (color-matched lines), mean radii of the cores and the core/shell structures are 2.0 and 2.9 nm, respectively. The corresponding standard deviations are 0.14 nm (7%) and 0.4 nm (14%), respectively. (c) XRD patterns of the starting ZB CdSe cores (blue) and core/shell CdSe/PbSe structures (red). Vertical bars show XRD peaks of bulk ZB CdSe (bottom, blue) and rock salt PbSe (top, red). (d,e) High-resolution TEM images of inverted CdSe/PbSe QDs prepared using ZB (d) and WZ (e) CdSe cores. Their mean total radii are 2.8 and 3.5 nm, respectively. From the combined analysis of the ICP-OES and TEM data (see text for details), the CdSe core radii for these QDs are 1.1 and 1.45 nm, respectively. Based on these values, the PbSe shell thicknesses are 1.7 nm (5.6 MLs) and 2.05 nm (6.7 MLs), respectively.

The analysis of the crystal structure using X-ray diffraction (XRD) indicates that the cores exhibit the expected features of a ZB CdSe crystal lattice (Figure 2c, blue trace). The XRD measurements of the core/shell QDs show the predominance of peaks characteristic of the PbSe rock-salt lattice (Figure 2c, red trace). This is a consequence of a larger volume of the PbSe shell compared to that of the CdSe core in the final core/shell structures.

In Figure S2, we show examples of TEM images and XRD patterns of the CdSe cores (*r* = 1.9 nm) and corresponding CdSe/PbSe structures (*R* = 3.5 nm) for samples grown using WZ cores. Both the core-only and core/shell QDs exhibit good size uniformity, comparable to that of the ZB-core samples. The XRD measurements show a transition from the WZ pattern for the core-only sample to the rock-salt pattern for the core/shell QDs. As indicated earlier, this occurs due to a larger volume of the PbSe shell compared to that of the CdSe core.

High-resolution TEM (HRTEM) images (Figure 2d,e) reveal a multifaceted structure of the core–shell interface, similar to that of conventional noninverted PbSe/CdSe QDs studied previously.^18^ Furthermore, the HRTEM images show that the shape of the core/shell QD replicates the shape of the original cores. In particular, the ZB CdSe cores exhibit a nearly spherical shape (Figure 2a) while the WZ cores are slightly elongated (Figure S2 and S3), as observed previously.^41^ These shapes are preserved following the formation of the CdSe/PbSe heterostructures (Figure 2d,e). This points toward highly uniform deposition of the shell material whose rate does not vary significantly between different nanocrystal facets. This is a direct consequence of our WL approach, which helps homogenize the core surface and, as a result, leads to good uniformity of the shell thickness.

Interesting information about the details of shell growth is provided by the comparison of TEM-based size measurements and the compositional analysis using inductively coupled plasma optical emission spectroscopy (ICP-OES) (Table S1). To discuss this comparison, we consider the case of the structures grown using WZ CdSe cores. As indicated earlier, the WZ core and resulting core/shell QDs have elongated shape. We define their size in terms of the effective (volume-equivalent) radius determined as *R* = (*a*^2^*b*)^1/3^, where *a* and *b* are the semimajor and semiminor axes, respectively. For the series of samples used in the ICP-OES studies, the CdSe core radius was 1.9 nm. After the preparation of a PbSe WL, the particle radius increased to ∼2.2 nm and then, to ∼2.9 nm following a continuing shell growth in a nonpolar medium using a 4 min reaction with Pb (II) oleate- and TOP-Se. The addition injection of the PbSe shell precursors produced the particles with *R* = 3.5 nm which completed the sample series.

Based on the ICP-OES measurements (Table S1), we can obtain the ratio of the numbers of the Pb (*N*_Pb_) and Cd (*N*_Cd_) ions (β = *N*_Pb_/*N*_Cd_) and then, use this quantity to calculate the core radius from *r* = *R*[1 + *β v*_PbSe_/(2*v*_CdSe_)]^−1/3^. Here *v*_CdSe_ and *v*_PbSe_ are the unit cell volumes of bulk WZ CdSe and rock-salt PbSe, respectively (*v*_CdSe_ = 0.112 nm^3^, *v*_PbSe_ = 0.236 nm^3^). A factor of 2 is added in front of *v*_CdSe_ because the rock-salt PbSe unit cell contains twice as many cations as the WZ CdSe unit cell. Using this expression, we obtain *r* of 1.9 to 2.0 nm for the PbSe shell growth time (*t*_sh_) of up to 3 min (*H* to about 0.7 nm), suggesting that the core retains its original size. For *t*_sh_ = 4 min, the CdSe core radius determined from ICP-OES measurements drops to 1.7 nm (*R* = 2.9 nm) and further to 1.45 nm following the additional injection of shell precursors (*R* = 3.5 nm). These observations suggest that the prolonged shell growth is accompanied by a partial Cd-for-Pb cation exchange, resulting in a gradual decrease in the CdSe core size.

For spectroscopic measurements, the QDs were purified using acetone as an aprotic solvent in air-free environment to reduce a detrimental effect of oxygen on PbSe-shelled QDs, redissolved in anhydrous toluene, and loaded into an optical cell with a thickness of 1 mm. Steady-state PL spectra were measured using low-intensity continuous wave (*cw*) excitation at 532 nm. Time-resolved PL measurements were performed using 100 fs pump pulses with a photon energy of 1.5 eV (NIR PL feature) or 3.1 eV (visible PL feature). To avoid complications caused by multiexciton effects, we used a low per-pulse fluence, corresponding to a sub-single-exciton average QD occupancy. The PL signal was time-resolved using a superconducting nanowire single-photon detector (SNSPD) with a temporal resolution of 60 ps.

Figures 3a and S3 show optical absorption and PL spectra of core-only and core/shell CdSe/PbSe QDs from the same series of samples that were used in the ICP-OES studies (Table S1). The original CdSe core particles (*r* = 1.9 nm) exhibit a sharp band-edge absorption peak at 576 nm and a fairly narrow PL band at 588 nm with a full width at half-maximum of 25 nm or 89 meV (Figure S3a, Supporting Information). After application of a WL, the particles become nonemissive but still exhibit a sharp band-edge absorption peak slightly shifted to longer wavelengths (the peak position is 584 nm; Figures 3a and S3a). This indicates the increase in the carrier effective localization volume due to expansion of carrier wave functions into the WL.

**Figure 3:**
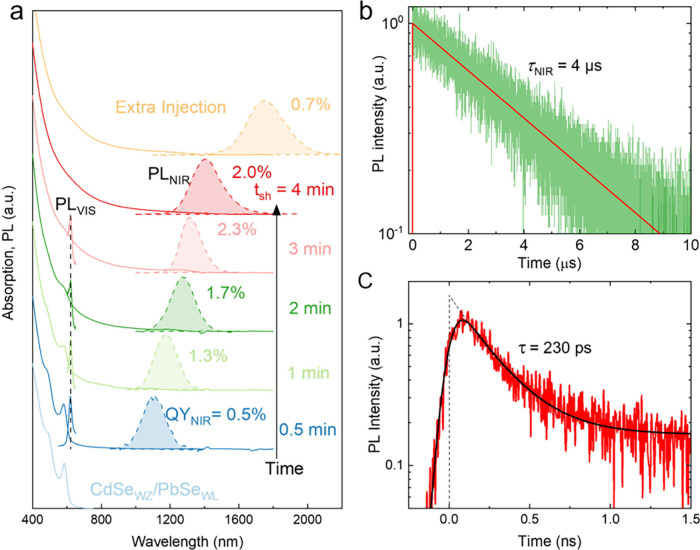
(a) Evolution of linear absorption and PL spectra of inverted core/shell CdSe/PbSe QDs with increasing shell thickness. These samples were prepared using WZ CdSe cores with *r* = 1.9 nm. The thickness of the PbSe shell (*H*) changes from 0.4 nm (shell growth time *t*_sh_ = 0.5 min) to 1.24 nm (*t*_sh_ = 4 min) and then to 2.05 nm (after extra injection of Pb and Se precursors) (b) PL dynamics of CdSe/PbSe QDs with *H* = 0.5 nm (*t*_sh_ = 1 min) measured at the peak (∼1200 nm) of the NIR band (green). Red line is a single exponential fit with a time constant of *τ*_NIR_ = 4 μs. (c) The visible PL dynamics measured at 619 nm (red line) is fitted to a double exponential decay (dashed black line) convolved with the instrument response function of the SNSPD apparatus, yielding the trace shown as the solid black line. Based on the fit, the initial (fast) relaxation time constant is 230 ps.

After particle transfer into the nonpolar phase followed by the 0.5 min shell-growth reaction (resulting in *H* of ∼0.4 nm), the QD sample exhibits dual-band PL consisting of a weak visible peak at 619 nm and a stronger NIR feature at *λ*_NIR_ = 1100 nm with a quantum yield (QY_NIR_) of ∼0.5% (Figure 3a). As indicated by our modeling of electronic states (discussed later in this work), these two features are due to a common band-edge electron state and two different hole states. The NIR band corresponds to a band-edge hole with preferential shell localization, while the visible band is due to a higher energy hole state which is primarily core localized. The absorption spectrum also shows signatures of core and shell components of the hetero-QDs. Specifically, it exhibits a structureless low-energy tail arising from the PbSe-shell-based states and the CdSe-core related peak at 584 nm.

For longer shell deposition times (*t*_sh_ = 1 to 3 min; the corresponding *H* is 0.5 to 0.7 nm), the CdSe core-related absorption feature becomes broader and less pronounced (Figure 3a). This is accompanied by a progressive increase of the intensity of the low-energy absorption tail arising from the PbSe shell. At the same time, the NIR PL band shifts toward lower energies (reaching 1400 nm for *t*_sh_ = 3 min) and increases in intensity (QY_NIR_ = 2.3% for *t*_sh_ = 3 min). Further increase of the shell thickness to 1.2 nm (*t*_sh_ = 4 min) and then to 2.05 nm (additional injection of shell precursors) leads to a continued redshift of the NIR PL band, but its intensity becomes weaker. For the thickest-shell sample (*H* = 2.05 nm, *R* = 3.5 nm), *λ*_NIR_ = 1750 nm and QY_NIR_ = 0.7%.

The change in the trend describing the dependence of QY_NIR_ on *H* correlates with the onset of the CdSe core etching due to the Cd-for-Pb cation exchange inferred from the ICP-OES studies (Table S1). This suggest that this process is accompanied by the formation of interfacial defects, which act as nonradiative decay centers. This also explains the complete quenching of visible PL at shell thicknesses greater than ∼1 nm (Figure 3a).

Time-resolved PL measurements reveal a dramatic difference in relaxation time scales of NIR and visible emission bands (Figure 3b,c). The NIR PL feature exhibits slow microsecond decay with a relaxation time constant of *τ*_NIR_ = 4 μs (Figure 3b), comparable to that of traditional NIR-emitting PbSe QDs and noninverted PbSe/CdSe core/shell structures.^19,42,43^ The visible band has much shorter decay. Using the trace obtained by the deconvolution of the measured PL dynamics with an instrument response function of our SNSPD detector, the visible PL relaxation time is *τ*_VIS_ = 230 ps (Figure 3c). This fast relaxation indicates the short-lived nature of at least one of the states (electron or hole) responsible for the visible emission. As mentioned earlier, this short-lived state likely corresponds to a higher-energy (hot) hole localized mainly in the CdSe core, implying that τ_VIS_ is determined by the relaxation of the hot hole to lower-energy states with preferential localization in the PbSe shell.

Interestingly, although the core-to-shell hole relaxation in our inverted core/shell structures is much faster compared to radiative decay, it is considerably slower than the intraband relaxation of hot carriers in standard core-only QDs. Indeed, typical intraband relaxation time constants in CdSe or PbSe QDs range from hundreds of femtoseconds to a few picoseconds.^44,45^ Since these time scales are orders of magnitude shorter than those of radiative decay, core-only QDs do not exhibit any discernible emission from hot carriers in excited states. On the other hand, our inverted core/shell QDs show a readily detectable hot PL which is a direct consequence of the rather slow hole transfer from core- to shell-based states. Below we discuss mechanisms responsible for the slowing down of hot hole relaxation in the inverted CdSe/PbSe core/shell QDs in the context of a theoretical analysis of their electronic states.

In our modeling, detailed in Supplementary Note 1, we use a mesh-based method for solving the radial Schrodinger equation in the single-band effective-mass approximation.^46^ In Figure 4a, we display the energy-level diagram of a core/shell structure with *r* = 1.9 nm and *R* = 2.9 nm, which corresponds to shell thickness *H* = 1 nm (approximately 3 PbSe MLs) along with energy levels of 1S_e,h_, 1P_e,h_, 1D_e,h_, and 2S_e,h_ electron and hole states.

**Figure 4:**
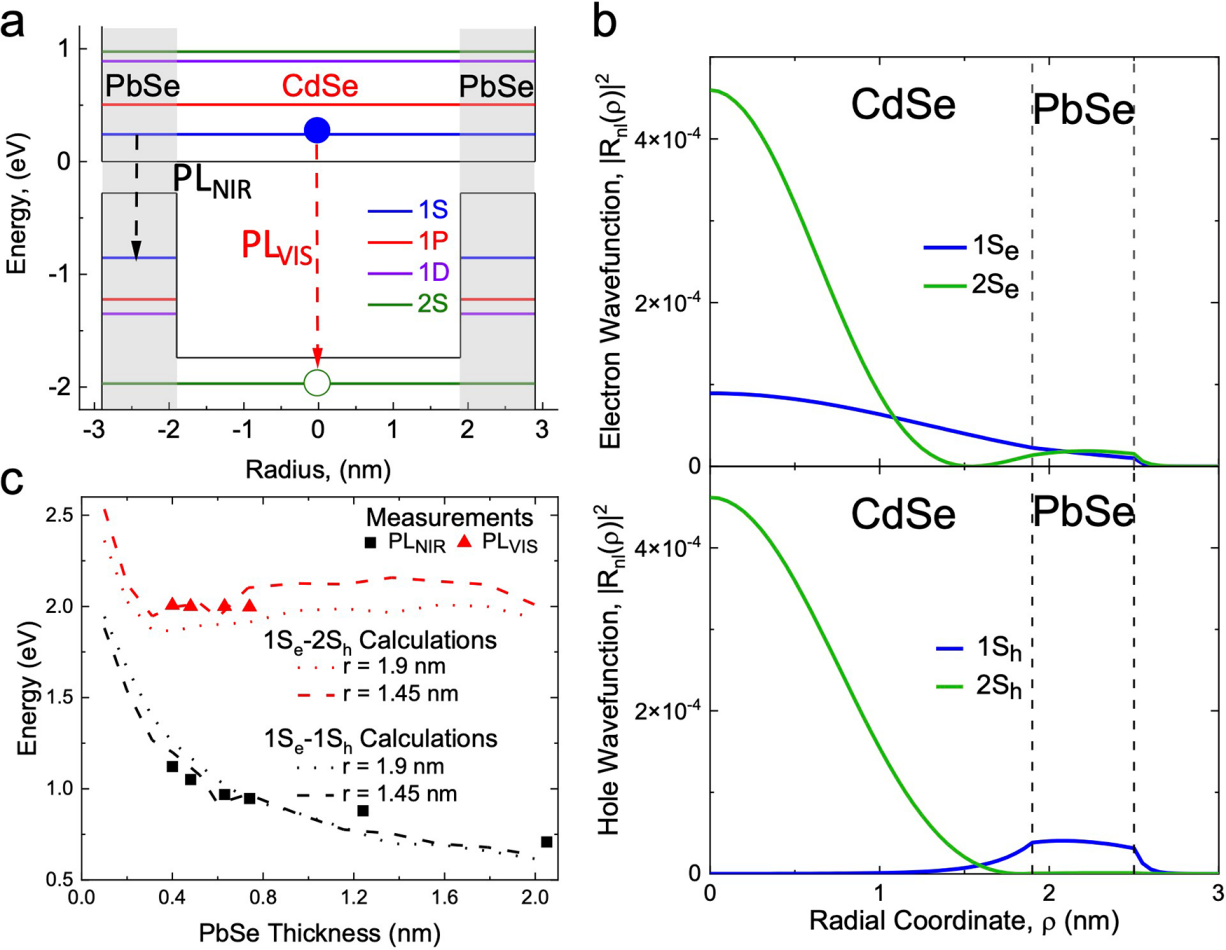
(a) Energy-level diagram of CdSe/PbSe QDs with *r* = 1.9 nm and *H* = 1 nm. The NIR emission band (PL_NIR_, dashed black arrow) is due to the delocalized 1S_e_ electron recombining with the shell-localized 1S_h_ hole, while the visible emission band (PL_VIS_, dashed red) is due to the delocalized 1S_e_ electron recombining with the core-localized 2S_h_ holes. (b) Calculated radial electron (top) and hole (bottom) wave functions for CdSe/PbSe QDs with *r* = 1.9 nm and *H* = 1 nm. The 1S_h_ and 2S_h_ states are localized preferentially within the shell and the core regions of the QD, respectively. (c) Calculated energies of the 1S_e_–1S_h_, 1S_e_–2S_h_ transitions (black and red lines, respectively) compared to the spectral energies of the NIR (black squares) and visible (red triangles) PL bands observed experimentally (Figure 3a). The core radii used in the calculations are *r* = 1.9 and 1.45 nm. These correspond to the maximum and minimum CdSe radius values found in Table 1. The shrinking CdSe radius is due to cation exchange during the growth of thicker PbSe shells.

Radial distributions of wave functions of two S-type electron and hole states (1S_e,h_, and 2S_e,h_) are shown in Figure 4b (the wave functions of the P- and D-type states can be found in Figure S4). Based on the calculations, all electron states are delocalized over the entire volume of a core/shell particle. In contrast, hole states show a pronounced difference in localization depending on their energy. In particular, the lowest energy 1S_h_ state is almost entirely shell localized and exhibits just weak leakage into the core region (Figure 4b, lower panel, blue line). Conversely, the higher-energy 2S_h_ state, located below the CdSe valence band-edge, is almost fully confined to the CdSe core (Figure 4b, lower panel, green line). As a result, the core- and shell-based hole states exhibit very weak wave function overlap. The other two shell-localized hole states (1P_h_ and 1D_h_) also exhibit very weak overlap with the 2S_h_ wave function (Figure S4). Together with the sizable energy gap between the 2S_h_ state and the nearest shell-based 1D_h_ level (Figure 4a), this impedes hole relaxation from the core to the shell and explains the emergence of narrow-band hot PL arising from the 1S_e_–2S_h_ transition.

The conducted calculations accurately describe the shell-thickness dependence of spectral energies of the observed NIR and visible PL bands (*E*_NIR_ and *E*_VIS_, respectively). In Figure 4c, we compare the measurements of *E*_NIR_ and *E*_vis_ (Figures 3a and S3) with modeling performed for the inverted core/shell QDs with *r* = 1.9 nm and *r* = 1.45 nm, and a varied shell thickness. The two radii used in the calculations are the maximum and minimum values found in Table S1. As discussed earlier, the reduction of the CdSe core radius observed for thicker shell samples is due to cation exchange during the latter stage of growth of the thicker PbSe shells. We observed a good overall agreement between our calculations and measurements for both the NIR and visible bands (Figure 4c). This, in particular, indicates that the visible PL is indeed due to the 1S_e_–2S_h_ transition, as suggested earlier.

The regime of slow carrier cooling is a useful property of the inverted core/shell CdSe/PbSe QDs which is sought for various advanced photoconversion schemes including up-conversion of NIR light,^26,47,48^ hot-carrier solar cells,^49^ and CM.^50−52^ However, traditional PbSe/CdSe QDs are not well suited for application in practical photoelectric devices or photochemistry. As discussed earlier, the primary problem is the difficulty to extract a band-edge hole which is confined to the PbSe core. This problem can be resolved with the inverted core/shell structures realized in the present study. As indicated by our modeling, in CdSe/PbSe QDs wave functions of 1S electrons and holes have a large amplitude at the QD surface (Figure 4b). Therefore, both types of carriers can readily communicate with the environment and, if necessary, be extracted from the QD and transported in the QD solid or the external circuit.

To elucidate the feasibilities of applications of the developed inverted QDs in photoelectric devices, we investigate transient photocurrent (TPC) in QD solid-state films using Auston switch devices employing a QD film as a photoactive layer^53−55^ (Experimental Section in the Supporting Information). These devices comprise a coplanar 50 Ω microstrip transmission line consisting of a gold layer (a ground plate) deposited onto the back side of a glass substrate and two top gold contacts separated by a 70 μm gap bridged by a film of QDs prepared via a layer-by-layer deposition approach (Figure 5a). To facilitate dot-to-dot charge transport, the QDs were cross-linked using solid-state ligand exchange to replace original long capping molecules with short bifunctional molecules of 1,2-ethanedithiol (EDT). These procedures are similar to those previously applied to fabricate Auston switches based on PbSe QDs.^54,55^

**Figure 5:**
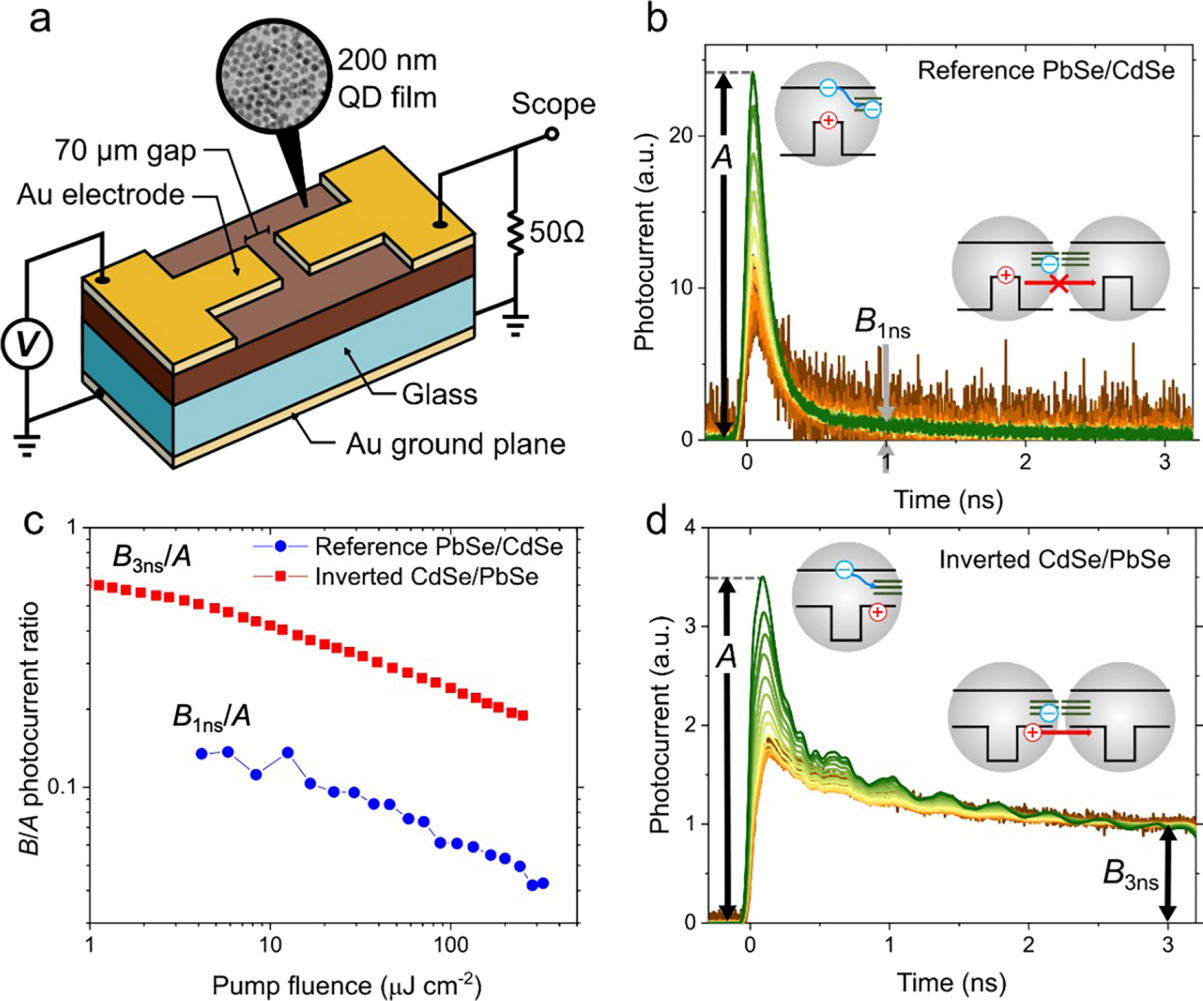
(a) A schematic depiction of a photoconductive Auston switch with a film of QDs as the photosensitive element. This device comprises a 200 nm thick QD film deposited onto a glass substrate with top gold electrodes that form a 50 Ohm transmission line together with a gold back plane. Photocurrent is generated in the 70 μm gap by short 110 fs laser pulses (1.2 eV photon energy) and monitored with a 20 GHz sampling oscilloscope. (b) Pump-power dependent tail-normalized TPC traces for EDT-treated reference (noninverted) PbSe/CdSe QDs (the total radius and shell thickness are ∼4 nm and ∼2.5 nm, respectively). The average per-dot excitonic occupancy ⟨*N*⟩ changes from 0.03 to 4. Following the pump pulse, the TPC signal quickly decays on a sub-ns time scale. This occurs due to fast trapping of photogenerated electrons by intragap states (upper inset). In these structures, photogenerated holes are immobile because of a large energy barrier created by the CdSe shell (lower inset). (c) Blue symbols show the ratio of the photocurrent measured at 1 ns after excitation (*B*_1 ns_) and the early time signal amplitude (*A*) for reference (noninverted) PbSe/CdSe QDs. Red symbols show a much higher ratio for inverted CdSe/PbSe QDs measured at 3 ns (see TPC traces in ‘d’). (d) Pump-power dependent tail-normalized TPC traces for EDT-treated inverted CdSe/PbSe QDs (ZB CdSe cores with *r* = 2 nm and shell thickness of 0.9 nm; same sample as in Figure 2a) for ⟨*N*⟩ ranging from 0.04 to 6. In addition to the initial fast component due to electron trapping (upper inset), the TPC traces contain a long-lived tail indicating dot-to-dot charge transport due to mobile holes (lower inset). The increase in the relative amplitude of the initial fast TPC component at high pump fluences (⟨*N*⟩ > 1) is due to nonradiative Auger recombination.

For TPC measurements, the device was biased using a 40-to-100 V voltage applied between one of the top contacts and the ground plane. The QDs in the gap region were excited by 110 fs pulses from an amplified femtosecond laser using a pump photon energy of 1.2 eV. With this photon energy, we excited only the PbSe component of the heterostructure to make sure that the detected photocurrent was not due to hot carriers in the CdSe region of the QD, but to band-edge carriers in the PbSe region. A TPC launched by the laser pulse was time-resolved with a 20-GHz oscilloscope used to monitor a transient voltage pulse generated on the 50 Ω resistor connected to the unbiased top contact and the ground plate (Figure 5a). The temporal resolution of this system is ∼50 ps.

First, we use the Auston-switch approach to investigate a TPC response of films made of traditional (noninverted) PbSe/CdSe QDs (Figure S6 and Experimental Section in the Supporting Information). In Figure 5b, we display TPC traces measured for a range of pump fluences that correspond to excitation of 0.06 to 4 excitons per dot per pulse on average (⟨*N*⟩). For all pump levels, the decay occurs on a short time scale of hundreds of picoseconds. Closer examination of photocurrent relaxation indicates that at low sub-single-exciton pump levels (⟨*N*⟩ < 0.1), the signal half-life is ∼170 ps and it shortens to ∼70 ps at the highest pump level (⟨*N*⟩ = 4). This shortening occurs due to the increasing fraction of the QDs populated with two or more excitons which decay via fast nonradiative Auger recombination.^54,56^

The low-pump-intensity TPC dynamics (⟨*N*⟩ < 0.1) observed for PbSe/CdSe QD samples are similar to the initial sub-ns photocurrent relaxation reported previously for plain, core-only PbSe QDs for which it was assigned to fast electron trapping by intragap states^55^ (Figure 5b, upper inset) forming a weakly conducting midgap band. This band is responsible for “dark” charge transport in nonilluminated films.^57^ It also plays an important role in photoconductance as it controls relaxation of photoinjected mobile carriers.^55^

According to studies of ref (55), in addition to the initial fast dynamics, core-only QD films exhibited a slow-relaxing component which was explained by nongeminate electron–hole recombination accompanying dot-to-dot migration of photoexcited holes. These mobile holes were identified as primary species responsible for photoconductance in PbSe QD films. The slow hole-transport-related dynamics are virtually absent in the films of reference (noninverted) PbSe/CdSe QDs (Figure 5b). In particular, the TPC signal measured at a time of Δ*t* = 1 ns after the pump pulse (*B*_1ns_) drops to ∼10% of the early time signal amplitude (*A*) and becomes undetectable at Δ*t* = 3 ns (Figure 5b,c, blue symbols). This is a direct consequence of core localization of photogenerated holes which inhibits their transport due to a high energetic barrier created by the CdSe shell (Figure 5b, lower inset).

The films of inverted CdSe/PbSe QDs exhibit a different behavior. In particular, in addition to fast (sub-ns) initial relaxation due to electron trapping (and Auger recombination at (⟨*N*⟩ > 1), they exhibit a pronounced slow-relaxing component (Figure 5d) in sharp contrast to the reference PbSe/CdSe QD sample. At low (sub-single-exciton) pump levels, the amplitude of the long-lived component measured at Δ*t* = 3 ns (*B*_3ns_) is approximately 70% of the peak photocurrent (Figure 5c, red symbols). This slow TPC component is similar to that observed in previous studies of device-grade core-only PbSe QDs^55^ (used, e.g., in PbSe QD solar cells^31^) where it was ascribed to charge transport dominated by mobile band-edge holes. This suggests that in films of the inverted CdSe/PbSe QDs, photogenerated holes are also mobile and, if necessary, can be accessed electrically (Figure 5d, lower inset). Thus, the developed inverted core/shell structures indeed exhibit the behavior we have aimed to achieve in the present work, and the films of these QDs are particularly applicable in photoelectric devices.

To summarize, we have developed a novel WL-based approach for uniform growth of a PbSe shell on top of CdSe cores. The synthesized core/shell QDs exhibit an inverted geometry due to which both an electron and a hole exhibit a significant degree of shell localization, which makes them accessible electrically. The developed structures are well suited for applications that require extraction of carriers from the QD (e.g., photochemistry) or carrier transport in the QD film (e.g., photovoltaics and photodetection). To illustrate the latter capability, we incorporate our inverted QDs into photoconductive Auston switches and use these devices to demonstrate that the photoexcited current exhibits a long-lived component due to the slow nongeminate recombination of mobile photocarriers.

The developed QDs are of considerable interest for implementing advanced photoconversion schemes exploiting effects such as hot carrier extraction^49,58^ and CM^24,59^ as well as their combination. Such scheme could benefit from slow relaxation of hot holes between the core and shell regions of the QD combined with the electrical accessibility of both band-edge carriers. In particular, standard noninverted PbSe/CdSe QDs have shown strong CM performance in all-optical spectroscopic measurements.^24^ The newly developed inverted QD are ideally suited for revisiting the CM topic using electro-optical or photochemical studies.

The developed approach may also simplify the realization of electronically and magnetically doped NIR QD materials. Their fabrication may benefit from multiple approaches to doping CdSe QDs with different impurities, the influence of which is expected to propagate to the PbSe shell after the preparation of inverted CdSe/PbSe hetero-QDs. This, in particular, may help advance the work on the recently discovered spin-exchange CM phenomenon^60^ from the initial purely optical studies of noninverted Mn-doped PbSe/CdSe QDs^60^ to device-relevant electro-optical studies of Mn-doped CdSe/PbSe structures with inverted geometry.

In addition, the WLAD synthesis protocol can be extended to create a wide range of other heterostructured QDs that are difficult to realize using existing synthetic methods. For example, traditional noninverted PbSe/CdSe QDs can be coated with a PbSe(S) outer shell to create structures with type (I+II) characteristics useful for applications as optical gain media.^61^ This will facilitate the development of NIR QD lasers and optical amplifiers applicable, for example, in telecommunications and silicon photonics. In addition, the developed WLAD approach can simplify the integration of dissimilar materials (e.g., semiconductors and metals) into a single nanocrystal, leading to mixed-functionality “hybrid” structures with interesting and potentially useful properties.

## Supporting Information Available

Details of the QD synthesis, microstructural characterization, optical spectroscopy studies, and fabrication and characterization of Auston switch devices (Experimental Section); theoretical modeling of QD electronic states (Supplementary Note 1); TEM and ICP-OES measurements (Table S1); schematic illustration of the shell growth process (Scheme S1); FTIR measurements (Figure S1); additional TEM and optical spectroscopy measurements (Figures S2 and S3); additional wave function and electronic energy calculations (Figures S4 and S5); optical absorption and PL spectra of noninverted PbSe/CdSe QDs (Figure S6); Supplementary References (PDF)
