# Supplementary material for: Inverted CdSe/PbSe Core/Shell Quantum Dots with Electrically Accessible Photocarriers
Source: ACS Energy Lett. 2025 Feb 5;10(2):1062–71. doi: 10.1021/acsenergylett.4c03502 (PMC11834150; doi:10.1021/acsenergylett.4c03502)
Supplement: Supplementary file 1 — nz4c03502_si_001.pdf [file nz4c03502_si_001.pdf]

Supporting Information for

Inverted CdSe/PbSe Core/Shell Quantum Dots with Electrically Accessible Photocarriers

Vladimir Sayevich, Whi Dong Kim, Zachary L. Robinson, Oleg V. Kozlov, Clément Livache, Namyoung Ahn, Heeyoung Jung, and Victor I. Klimov*

Nanotechnology and Advanced Spectroscopy Team, C-PCS, Chemistry Division, Los Alamos National Laboratory, Los Alamos, New Mexico 87545, United States

Email: klimov@lanl.gov

Experimental Section

Materials: Lead (II) oxide (PbO, 99.999%, Aldrich), lead acetate trihydrate (≥99.99%, Aldrich), oleic acid (OA, 90%, Aldrich), trioctylphosphine (TOP, 97%, STREM), oleylamine (OLA, 70%, Aldrich), selenium powder (Se, 200 mesh, 99.999%, Alfa Aesar), trioctylamine (98%, Aldrich), cadmium oxide (CdO, 99.99%, Aldrich), cadmium chloride ($CdCl_2$, 99.99%, ACROS Organics), sodium selenide (Na_2Se, 99.8%, Alfa Aesar), *N*-octadecylphosphonic acid (ODPA, >97%, Aldrich), cadmium nitrate tetrahydrate (99.999%, Aldrich), myristic acid (98.5%, Aldrich), selenium dioxide (SeO_2, 99.999%, Aldrich), 1,2-hexadecandiol (90%, Aldrich), octadecene (ODE, 90%, Aldrich), *N*-methylformamide (MFA, 99%, Aldrich), *N*-butanol (99.8%, Aldrich) and other solvents and non-solvents were used as received. Octadecene and MFA were dried at 80° C for 10 hours under vacuum and stored in a glovebox.

***Preparation of CdSe cores*:** Monodisperse CdSe quantum dots (QDs) with the wurtzite (WZ) crystal structure in the size range of 2–4 nm were synthesized following ref. [1]. Synthesis of sinc blende (ZB) CdSe QDs in a size range of 3–4 nm was performed *via* a previously described method of ref. [2]. After the synthesis, the QDs were precipitated with either acetone or *N*-butanol (one time) and ethanol (twice) and redispersed in hexane with a concentration of ~150 mg mL^{-1}. The concentration was calculated based on the measured absorption coefficient and QD extinction coefficients from ref. [3].

***Preparation of a wetting layer*:** All synthetic steps were performed in a glove-box under oxygen-free and moisture-free conditions using c-ALD at room temperature. Original organic ligands of the CdSe QDs (20–50 mg in 3 mL of hexane) were replaced with Se^{2-} ionic species (0.1 M solution of Na_2Se in 3 mL of MFA) using a solution-phase ligand exchange reaction leading to the formation of a stable colloid of the Se^{2-}-capped CdSe QDs. The Se^{2-}-capped CdSe QDs were precipitated twice using MFA–acetone/toluene as a solvent–non-solvent pair to remove the unreacted precursors and remaining reaction byproducts. Afterward, the QDs were transferred to polar MFA for growth of the Pb half-layer of a WL. 0.1–0.2 mL of 0.5 M Pb^{2+} cation precursor solution (in the form of lead-acetate in MFA) was added to diluted Se^{2-}-capped CdSe QDs (3–6 mg in 5 mL of MFA), and the mixture was stirred for 2–3 min. It is important to use diluted Se^{2-}-capped CdSe QDs to prevent flocculation or gelation of the QDs due to the collapse of the diffuse counterion cloud around the electrostatically stabilized QDs. The CdSe/Se^{2-}/Pb^{2+} QDs consisting of a CdSe cores and a thin PbSe layer were precipitated one time using MFA–acetone/toluene as a solvent–non-solvent pair. These procedures produced a stable solution of the CdSe/Se^{2-}/Pb^{2+} QDs in MFA with a concentration of ~60 mg mL^{-1}.

***Transfer of* $CdSe/Se^{2-}/Pb^{2+}$ QDs *into a non-polar solvent*:** The next step was the surface functionalization of $CdSe/Se^{2-}/Pb^{2+}$ QDs with oleate ions (introduced as lead-oleate) to assist the transfer of the QDs from polar MFA into a non-polar medium. The oleate solution was prepared by dissolving 953.6 mg of PbO and 3.5 mL of oleic acid in 20 mL of ODE, degassed by heating to 110 °C under vacuum and then, under nitrogen at 150 °C for 1 and 2 hours, respectively. 3 mL of the stock oleate solution in ODE was added to dry $CdSe/Se^{2-}/Pb^{2+}$ QDs, which were precipitated from MFA. After mild ultrasonification, the QDs were transferred to ODE, leading to the formation of oleate-capped $CdSe/Se^{2-}/Pb^{2+}$ QDs. These structures are referred to in this work as functionalized (CdSe+WL) cores. The QDs were flocculated with ethanol and redispersed in ODE to form a stable concentrated solution (30–60 mg mL^{-1}).

***High-temperature PbSe shell growth*:** To grow a PbSe shell, lead and selenium precursors were prepared in advance. The lead-precursor solution was prepared by dissolving 444.2 mg of PbO in 2 mL of oleic acid and 10 mL of ODE at 150 °C under nitrogen flow (for 2 hours) and subsequently dried under vacuum at 110 °C (for 1 hour). The selenium-precursor solution was prepared by dissolving 1.5 mL of TOP·Se (1 M) in 4 mL of TOP.

To prepare a PbSe shell with a thickness of up to 3 semiconductor monolayers (MLs) on top of ZB CdSe cores with a radius of 1.9 nm, 700 µL of (CdSe+WL) seeds with a concentration of $5 \cdot 10^{-4}$ M in ODE in one syringe and a solution of 1.5 mL of 1 M TOP·Se in 4 mL of TOP in another syringe were simultaneously injected into the dried solution of 9 mL of lead-precursor, 16 mL of ODE and 4 mL of oleic acid at 130 °C. The temperature of the reaction mixture was increased up to 150 °C within 3 mins followed by fast cooling with a water bath.

To grow a PbSe shell up to a 4-ML thickness on top of WZ CdSe cores with a radius of 1.9 nm, 1 mL of freshly prepared (CdSe+WL) seeds with a concentration of $5 \cdot 10^{-4}$ M in ODE in one syringe

and a solution of 1.5 mL of 1 M TOP·Se in 4 mL of TOP in a separate syringe were injected into the dried solution of 9 mL of lead-precursor, 16 mL of ODE, and 4 mL of oleic acid at 130 °C. The temperature of the reaction mixture was increased up to 160 °C within 4 mins followed by fast cooling with a water bath.

To grow thicker shells ($H > 3$ ML for ZB cores, and $H > 4$ ML for WZ cores), multiple injections of small amounts of lead- and selenium-precursors were performed at 140 °C within 10 min. The synthesized core/shell QDs were precipitated from solution by adding a minimum amount of ethanol-acetone (1/3 V/V). The QDs were redispersed in toluene and stored in a nitrogen glovebox.

***Synthesis of noninverted core/shell PbSe/CdSe QDs*:** First we prepare PbSe QDs using the following approach._ 476.8 mg of PbO and 2.74 mL of oleic acid were dissolved in 15 mL of ODE. The mixture was heated to 150 °C for 0.5 h under nitrogen to form a clear solution of lead (II) oleate and degassed at 100 °C under vacuum (~10^{-2} bar) for 1 h. Then, the resulting solution was heated to 170 °C. and a mixture of 6.75 mL of 1 M TOP·Se and 35 μL of diphenylphosphine was quickly injected into it. The PbSe QDs were grown for 2 min followed by an abrupt cooling using ice-water. To precipitate the synthesized particles, ethanol was added to the solution. The colloid was further centrifuged, and the precipitate was redispersed in pure hexane. This cleaning step was repeated twice. Finally, the PbSe QDs capped with oleic acid were redispersed in octane and stored in a glovebox.

To prepare core/shell PbSe/CdSe QDs, we use cation exchange of Pb^{2+} for Cd^{2+} withint the surface layer of the synthesized PbSe QDs. To prepare Cd-oleate, a mixture of 0.64 g of CdO, 5 mL of oleic acid, and 5 mL of ODE was heated to 260 °C and kept at this temperature for 1 hr. The resulting solution was placed under vacuum at 110 °C for 1 hour. PbSe QDs in hexane were slowly added into the cadmium-oleate mixture at 60 °C, and then hexane was completely removed under

vacuum. A cation exchange reaction was conducted at 120 °C for 16 hrs to form PbSe/CdSe QDs. The washing of PbSe/CdSe QDs was performed twice using ethanol-toluene as a solvent/non-solvent pair.

***Structural and compositional studies of QDs*:** Transmission electron microscope (TEM) and high-resolution TEM (HRTEM) images were taken using a JEOL 2010 TEM. QD samples were prepared as drop-cast films deposited onto carbon-coated copper grids.

Powder X-ray diffraction (XRD) patterns were collected with a PANalytical EMPRYREAN diffraction system in the reflection mode. A nickel filter, Cu Kα1 irradiation, and a PIXcel 1D detector were used in the measurements.

The chemical composition of the QDs was determined using a Shimadzu ICPE-9000 ICP-OES system.

***Sample preparation for spectroscopic measurements*:** During sample preparation, QDs were handled in a nitrogen glove box to avoid oxidation. The purified QDs dispersed in trichloroethylene (TCE) were loaded into a 1 mm thick air-tight quartz cuvette and tightly sealed. QD samples were diluted such that to obtain an optical density of ~0.05 at the position of the band-edge transition. During time-resolved photoluminescence (PL) measurements, the QD solution was continuously stirred to avoid uncontrolled photocharging and sample degradation.

***Optical absorption and steady state PL measurements*:** Absorption spectra were measured by a PerkinElmer Lambda 950 spectrophotometer. Visible PL spectra were recorded using a Horiba Scientific FluoroMax-4 spectrofluorometer with a 400 nm excitation source. NIR PL spectra were taken using a home-built system with a 532 nm laser as a source of excitation. QD emission was analyzed by scanning a grating monochromator coupled to a liquid nitrogen cooled InSb detector.

A near infrared (NIR) PL quantum yield (QY) was obtained using IR-26 dye (QY = 0.048%) as a reference.

***Time-resolved PL measurements*:** The QD samples were excited using ~100 fs pulses of either the fundamental output (1.55 eV photon energy; NIR PL measurements) or the second harmonic output (3.1 eV photon energy; visible PL measurements) of a femtosecond Ti:sapphire amplified laser (Coherent RegA 9000). The QD emission was spectrally filtered using a monochromator and detected by a superconducting single-nanowire single-photon detector (SNSPD) cooled to 3.5 K. A width of the instrument response function of this system was ~60 ps.

***Fabrication of Auston switch devices*:** 100 nm of gold was evaporated onto the back of a glass substrate. A 200-nm thick QD film (inverted CdSe/PbSe or noninverted PbSe/CdSe QDs) was deposited on top of the substrate *via* repeated spin-coating/ligand exchange/rinsing steps. During the deposition procedure, the QDs dispersed in octane (20 mg mL^{-1}) were spin-coated at 1600 rpm for 30 s. For ligand exchange, 60 μL of a 1 M EDT solution in MeOH was dropped onto the QD layer, soaked for 60 s and then, spin-coated at 1200 rpm for 30 s. For rinsing, pure MeOH was spin-coated onto the QD layer to remove residual excess ligands. To prepare a 200-nm thick QD layer, these three steps were repeated 5 times. The resulting QD film was annealed for 30 min at 150 ^{o}C. Two gold contacts (200 nm-thickness) separated by a 70 μm gap were evaporated on top of the QD film through a shadow mask. Together with the gold plate at the bottom of the substrate, these contacts form a co-planar microstrip transmission line with nominal impedance of 50 Ω.

***Transient photocurrent measurement*:** The as-prepared devices were loaded into a nitrogen-filled air-tight housing to avoid QD oxidation. One of the top gold contacts was connected using coaxial cables to a sampling oscilloscope with a 20 GHz bandwidth, while the other contact was biased using a direct-current voltage source. QDs in the gap between the gold contacts were excited by

110-fs, 1.2-eV pulses of a regeneratively amplified ytterbium-gadolinium tungstate (Yb:KGW) femtosecond laser (Pharos, Light Conversion). The pulse repetition rate was 20 kHz. The laser beam was focused with a cylindrical achromatic lens into a stripe with dimensions of ~2 mm by ~300 μm, which were much larger than the device photoactive area (500 μm by 70 μm) and, as a result, allowed us to achieve spatially uniform excitation. To minimize timing jitter, the oscilloscope was triggered by laser pulses using a high-speed Si photodetector.

Supplementary Note 1: Modeling of electronic states

In our modeling, we use a mesh-based method for solving the radial Schrodinger equation in the single-band effective-mass approximation taking into account the electron-hole Coulomb interation.[4] We consider heterostructures with a WZ CdSe core and use bulk semiconductor bandgaps (E_g) of 1.74 eV (CdSe) and 0.28 eV (PbSe), and carrier effective masses of $0.13m_0$ (electrons) and $0.45m_0$ (holes) for CdSe, and $0.084m_0$ (electrons) and $0.07m_0$, (holes) for PbSe. Coulomb interactions were included using dielectric constants of 6.3 for CdSe, 22.9 for PbSe, and 1 for the external medium. We further assume that the conduction-band energy offset (ΔE_{CB}) at the CdSe/PbSe is zero,[5] implying that the valence-band energy offset (ΔE_{VB}) is 1.46 eV ($\Delta E_{VB} = E_{g,CdSe} - E_{g,PbSe}$). The QD states are labeled as '*nl*'. Here *l* is the orbital momentum of the envelope wavefunction denoted as S, P, and D for states with $l = 0$, 1, and 2, respectively, and *n* is the state number in a series of states with the same *l*, which varies from 1 to infinity.

We would like to point out that the inclusion of the Coulomb interaction was necessary to obtain an accurate description of the experimental observations as illustrated in Figure S5. Otherwise, the calculated transition energies were higher than the observed PL energies. Since the electron-hole interaction is attractive, it reduces the energy of the interband transitions, which leads to the

improved agreement with the measurements. The Coulomb correction is ~400 meV for a thin PbSe shell of 1 ML thickness and decreases to ~170 meV for a 7 ML shell (in both cases r = 1.9 nm).

Table S1. TEM and ICP-OES measurements of a series of inverted core/shell CdSe/PbSe QD samples with different PbSe shell thicknesses. These samples were prepared using WZ CdSe cores with r = 1.9 nm. N_{Pb} and N_{Cd} are the numbers of the Pb and Cd ions in QD, respectively.

Shell growth time (min)	$\beta = N_{Pb}/N_{Cd}$ (ICP-OES)	QD total radius (nm) (TEM)	CdSe core radius* (nm)	PbSe shell thickness** (nm)
0			1.9	0
0.5	0.71±0.05	2.3±0.3	1.9	0.4
1	0.85±0.06	2.5±0.2	2.0	0.5
2	1.22±0.09	2.6±0.2	2.0	0.6
3	1.52±0.11	2.7±0.2	2.0	0.7
4	4.1±0.3	2.9±0.2	1.7	1.2
Additional injection	12.3±0.9	3.5±0.3	1.45	2.05

*CdSe core radius (r) was calculated using $r = R[1 + \beta\, v_{PbSe}/(2v_{CdSe})]^{-1/3}$, where R is the total QD radius, and v_{PbSe} and v_{CdSe} are the unit cell volumes of bulk WZ CdSe and rock-salt PbSe, respectively.

**PbSe shell thickness (H) was obtained from $H = R - r$.

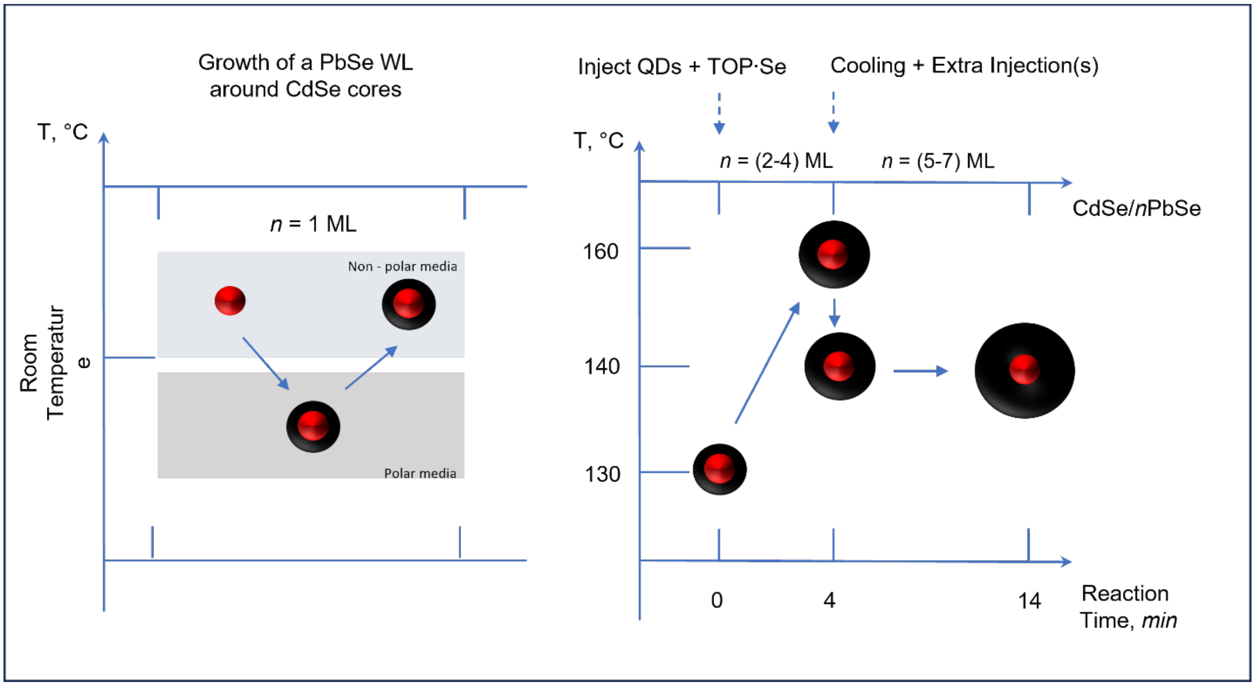

Scheme S1. Schematic illustration of the shell growth process. Starting with CdSe cores at room temperature, original organic ligands are replaced with Se^{2-} ionic species during quantum dot (QD) transfer from a non-polar medium (ODE) to a polar solvent (MFA) using solution-phase ligand exchange. Then colloidal atomic layer deposition (c-ALD) is applied to deposit a layer of Pb^{2+} cations, which completes a Se-Pb wetting layer (WL). After addition of lead-oleate, the CdSe/Se^{2-}/Pb^{2+} QDs are transferred back to a non-polar medium (ODE) to form oleate-capped CdSe/Se^{2-}/Pb^{2+} QDs referred here to as 'functionalized (CdSe+WL) cores.' To grow the PbSe shell, the (CdSe+WL) particles and Pb and Se precursors are injected into a mixture of ODE and oleic acid at 130°C. The temperature of the reaction is increased to 160°C within several minutes which leads to shell thicknesses from 2 to 4 PbSe monolayers (MLs). The shell thickness can be further increased to 5–7 MLs by fast cooling to 140°C followed by extra injections of Pb and Se precursors.

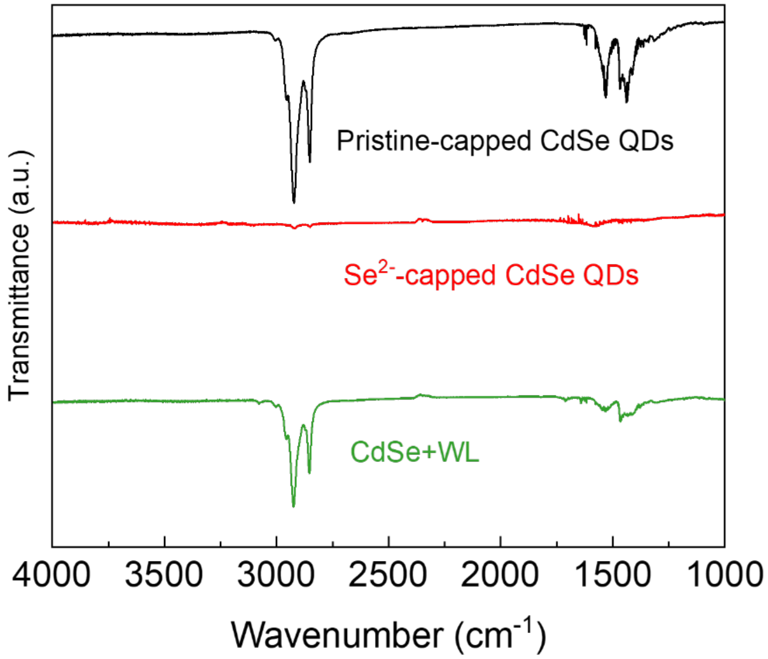

Figure S1. Fourier transform infrared (FTIR) spectra of WZ core-only CdSe QDs, with a radius of r = 1.9 nm capped with original organic ligands (black trace) and after ligand exchange for inorganic Se^{2-} species and transfer into a polar phase (red trace). Green trace is the FTIR spectrum of QDs enclosed into a PbSe WL (CdSe+WL) and transferred back into a nonpolar phase with oleate co-ligands. The spectra are offset vertically for clarity. The characteristic C-H stretching modes (2700–3000 cm^{-1}) completely disappear following ligand exchange with Se^{2-} species, confirming a complete removal of original organic capping groups. The C-H stretching modes re-appear after the (CdSe+WL) particles are transferred back into a nonpolar solvent.

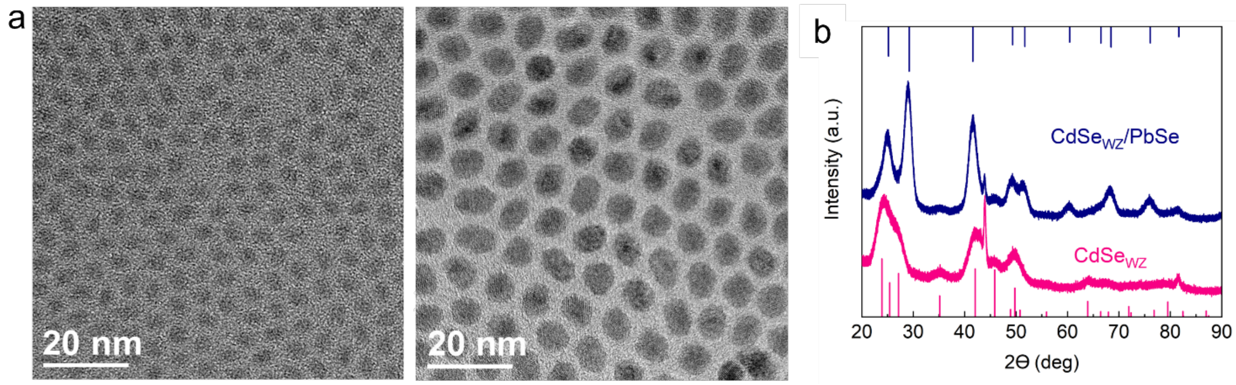

Figure S2. (a) Transmission electron microscopy (TEM) images of starting wurtzite (WZ) CdSe cores with a radius of $r = 1.9$ nm (left) and final CdSe/PbSe core/shell QDs (right) with a PbSe shell thickness (H) of approximately 2 nm or ~7 semiconductor MLs. The overall radius of the core/shell QDs (R) is approximately 3.5 nm. (b) The corresponding X-ray diffraction patterns of the core (pink) and the core/shell (blue) samples. Vertical bars show positions of diffraction peaks of bulk WZ CdSe (bottom; pink) and bulk rock salt PbSe (top; blue).

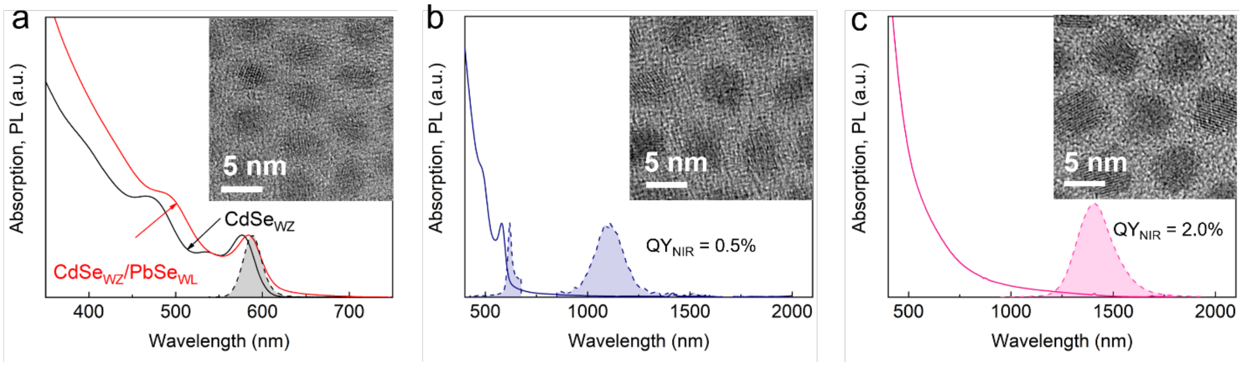

Figure S3. Linear absorption (lines), steady-state photoluminescence (PL, shaded spectra), and corresponding high-resolution TEM images (insets) of (a) WZ CdSe cores (black lines) with a radius of $r = 1.9 \pm 0.2$ nm, (b) CdSe/PbSe QDs for a shell-growth time of $t_{sh} = 0.5$ min ($H = 0.4$ nm or ~1.3 ML), and (c) $t_{sh} = 4$ min ($H = 1.2$ nm or ~4 MLs). QY_{NIR} is the quantum yield of the near-infrared (NIR) PL band. The red line in (a) is the absorption spectrum of non-emissive CdSe/PbSe QDs which contain a 1 ML thick PbSe wetting layer (WL).

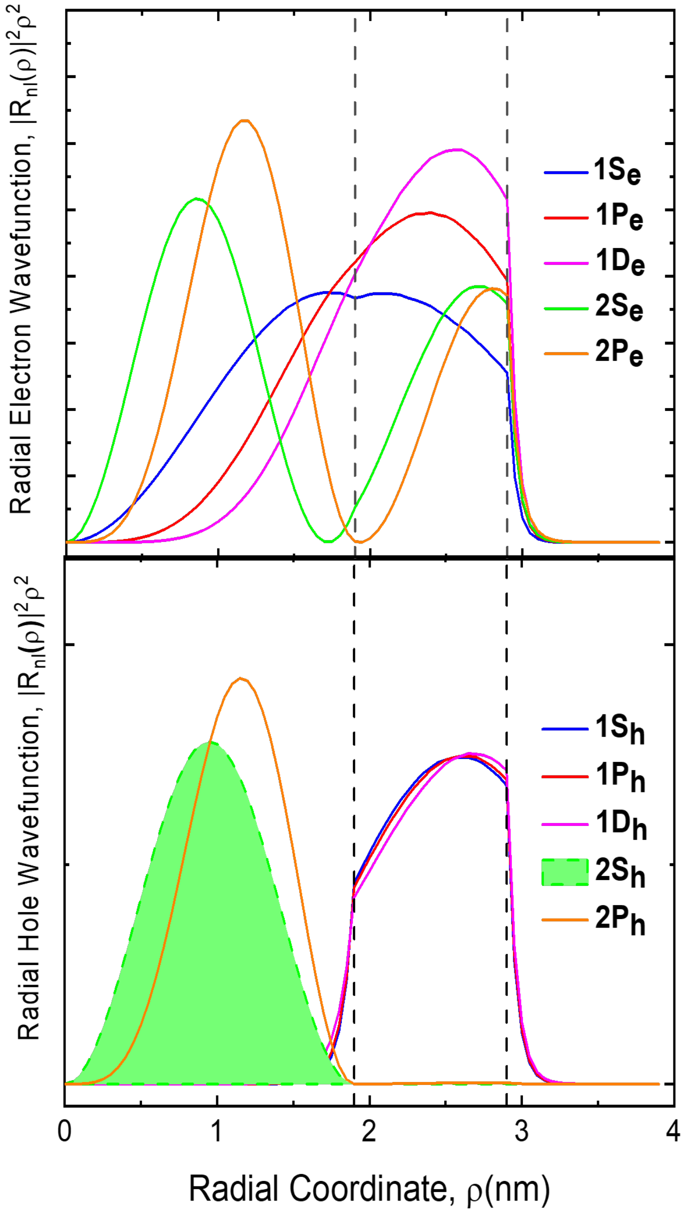

Figure S4. Calculated radial electron and hole wavefunctions ($R_{nl}(\rho)$) for five lower-energy electron and hole states ($1S_{e,h}$, $1P_{e,h}$, $1D_{e,h}$, $2S_{e,h}$, and $2P_{e,h}$) in inverted CdSe/PbSe QDs with r = 1.9 nm and H = 1 nm.

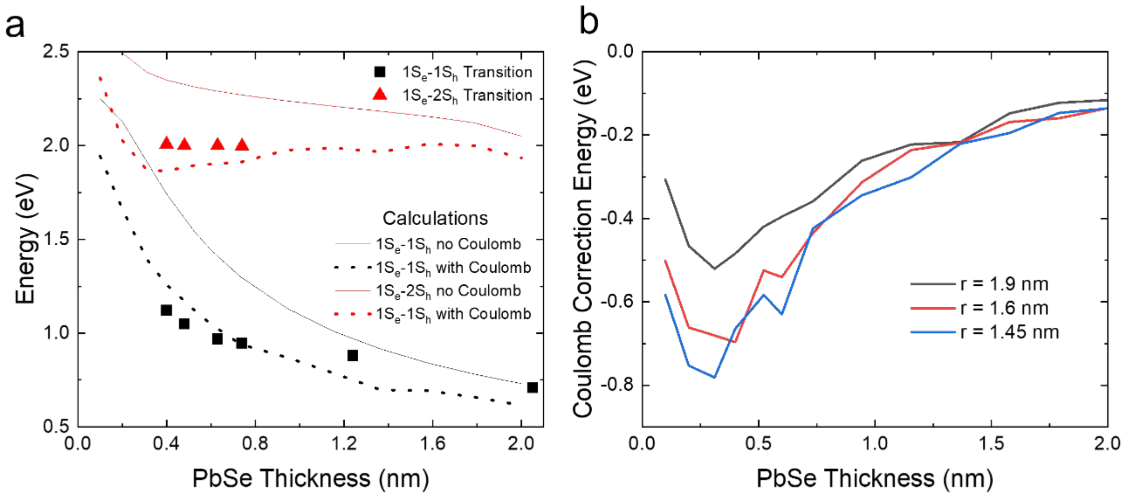

Figure S5. (a) Calculated $1S_e$–$1S_h$ (black) and $1S_e$–$2S_h$ (red) transition energies as a function of shell thickness for r = 1.9 nm without accounting for Coulomb interactions (solid lines) and with the electron-hole Coulomb interaction included (dashed lines) as is shown in the main text. Symbols are the spectral energies of the visible (red triangles) and NIR (black squares) PL bands from Figure 3. (b) The electron-hole Coulomb interaction energy for three different core radii (r = 1.45, 1.6, and 1.9 nm). The oscillations come from imposing a finite grid on sub-ML spacings. Decrease in the magnitude of the Coulomb correction for small PbSe shell thicknesses (<0.3 nm) is due to weak confinement of holes in a sub-ML PbSe layer.

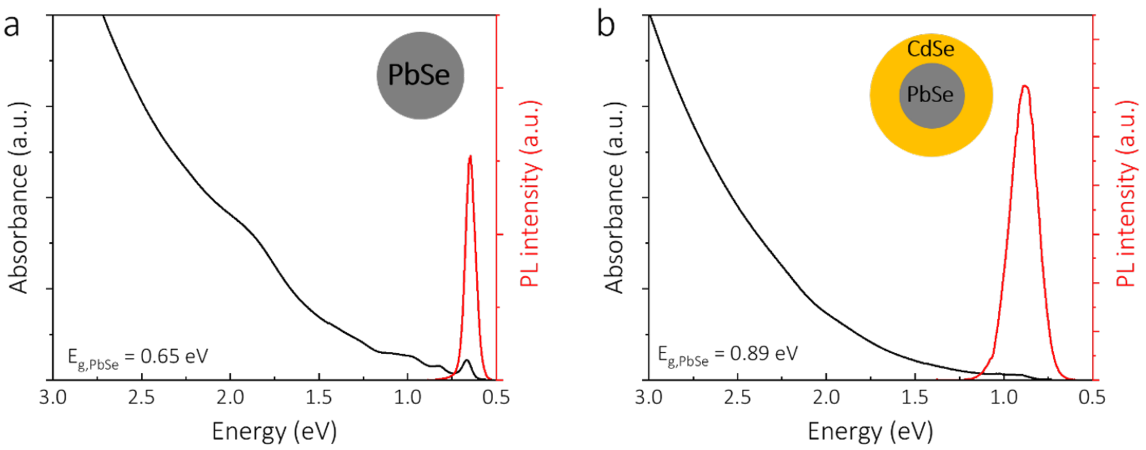

Figure S6. PL (red line) and absorption (black line) spectra of PbSe cores (a) and noninverted PbSe/CdSe core/shell QDs (b). The overall QD radius is ~4 nm and the shell thickness is ~2.5 nm. These core/shell QDs were used as the reference sample in TPC studies shown in Figure 5.

Supplementary References

1 Carbone, L., Nobile, C., De Giorgi, M., Sala, F. D., Morello, G., Pompa, P., Hytch, M., Snoeck, E., Fiore, A., Franchini, I. R., Nadasan, M., Silvestre, A. F., Chiodo, L., Kudera, S., Cingolani, R., Krahne, R. & Manna, L. Synthesis and micrometer-scale assembly of colloidal CdSe/CdS nanorods prepared by a seeded growth approach. *Nano Lett.* **7**, 2942-2950 (2007).

2 Chen, O., Chen, X., Yang, Y., Lynch, J., Wu, H., Zhuang, J. & Cao, Y. C. Synthesis of Metal–Selenide Nanocrystals Using Selenium Dioxide as the Selenium Precursor. *Angew. Chem. Int. Ed.* **47**, 8638-8641 (2008).

3 Jasieniak, J., Smith, L., van Embden, J., Mulvaney, P. & Califano, M. Re-examination of the Size-Dependent Absorption Properties of CdSe Quantum Dots. *J. Phys. Chem. C* **113**, 19468-19474 (2009).

4 Lin, Q., Makarov, N. S., Koh, W.-k., Velizhanin, K. A., Cirloganu, C. M., Luo, H., Klimov, V. I. & Pietryga, J. M. Design and Synthesis of Heterostructured Quantum Dots with Dual Emission in the Visible and Infrared. *ACS Nano* **9**, 539-547 (2015).

5 Madelung, O. *Semiconductors: Data Handbook, 3rd Edition*. (Springer-Verlag, 2004).
